# Editorial: Coping with antimicrobial resistance in the context of dental medicine

**DOI:** 10.3389/fdmed.2026.1807351

**Published:** 2026-03-04

**Authors:** Lucinda J. Bessa, Simonetta D’Ercole, Ricardo Alves

**Affiliations:** 1 Egas Moniz Center for Interdisciplinary Research (CiiEM), Egas Moniz School of Health & Science, Almada, Portugal; 2 Department of Medical Oral and Biotechnological Sciences, University “G. D’Annunzio” Chieti-Pescara, Chieti, Italy

**Keywords:** antibiotic stewardship, antimicrobial resistance, combined therapies, dental medicine, prescribing practices, quorum quenching

## 1 Introduction

Antimicrobial resistance (AMR) stands among the foremost threats to global public health in the 21st century, compromising our ability to treat infectious diseases and threatening decades of antibiotic-driven therapeutic advancement. The World Health Organization consistently ranks AMR as a top global health threat due to its contribution to increased morbidity, mortality, and economic burden across healthcare systems worldwide (1). Combatting AMR requires a One Health approach, integrating human, animal, and environmental health to address the drivers and consequences of resistance. Within this framework, the field of dental medicine holds a pivotal but often under-recognized role: dentists and dental professionals are responsible for approximately 10% of all antibiotic prescriptions in human medicine, making stewardship and education in dental settings essential for mitigating inappropriate antimicrobial use (2–4). In particular, AMR triggering factors in dentistry are: inappropriate prescription, self-medication and non-adherence to therapy, unnecessary prophylaxis (5).

This Research Topic — *Coping with Antimicrobial Resistance in the Context of Dental Medicine* — brings together a collection of multidisciplinary contributions that explore diverse facets of AMR relevant to dentistry. These include empirical investigations into prescribing practices, microbiological research on resistant pathogens, and reviews of potential alternative therapeutic strategies. Collectively, the articles advance our understanding of how AMR manifests in dental practice and highlight opportunities for interventions that support responsible antibiotic use, improved clinical outcomes, and reduced resistance selection.

## 2 Overview of contributing articles

### 2.1 Antibiotic prescribing practices and stewardship education

The cross-sectional original research article by Danadneh et al*.* examines antibiotic resistance knowledge, attitudes, and prescribing behaviors among dental students in Palestine. Through a structured survey, the study highlights significant gaps between students’ theoretical understanding of antibiotic use and their readiness to apply stewardship principles in clinical practice. Although foundational knowledge about AMR was relatively high, a substantial proportion of participants reported inappropriate prescribing behaviors—often influenced by diagnostic uncertainty, time pressures, and perceived patient expectations. Importantly, greater access to guidelines, higher self-confidence, and more positive attitudes toward stewardship were associated with lower prescribing frequency, underscoring the need to embed antibiotic stewardship more effectively into dental curricula and clinical training.

### 2.2 Synergistic approaches to antifungal therapy

The original research by Aliaghazadeh et al*.* explores the synergistic effects of icariin,a flavonoid found in plants of the genus *Epimedium,* combined with azole antifungal drugs against *Candida albicans*. This study provides insight into alternative strategies that may reduce reliance on traditional antifungals alone and presents mechanistic evidence for combined regimens that retain efficacy against resistant fungal isolates. These findings have potential implications for managing oral fungal infections in settings where resistance limits therapeutic options.

### 2.3 Natural products and quorum-quenching potential

The mini-review by Hashim et al*.* considers Gum Arabic as a potential quorum-quenching agent in periodontal therapy. Quorum sensing—the microbial communication that coordinates biofilm formation and virulence—plays a key role in periodontal disease. Targeting microbial communication rather than directly killing bacteria could reduce the selective pressures that drive resistance, suggesting a promising area for adjunctive or alternative interventions within dental practice.

### 2.4 Surveillance of resistant oral pathogens

An original research study by Faustova et al. employs cluster analysis to identify antifungal drugs that retain efficacy against clinical *C. albicans* isolates from patients with maxillofacial soft-tissue inflammatory diseases. Such surveillance work is crucial for characterizing resistance patterns among clinically relevant pathogens and informing empiric therapy decisions in contexts where traditional susceptibility data may be limited.

### 2.5 Antimicrobial resistance among gram-negative bacilli

A retrospective study by Basic et al*.* assesses antibiotic resistance profiles of aerobic Gram-negative bacilli isolated from patients with oral inflammatory dysbiotic conditions. By delineating resistance trends within these opportunistic pathogens, this work contributes valuable data to local and regional susceptibility maps, which are essential for guiding empiric antimicrobial choices and stewardship policies in dental and oral healthcare settings.

## 3 Discussion and future directions

The contributions to this Research Topic collectively underscore the complexity of addressing AMR within dental medicine. From surveillance and microbiological evaluation to education and stewardship practice, the articles reflect a continuum of research that is essential for developing evidence-based interventions. Key themes include the alignment of knowledge and clinical practice, the integration of alternative therapeutic strategies, and the importance of standardized guidelines and access to stewardship resources.

Central to progress in this field is educational reform: dental students and practitioners must not only understand AMR conceptually, but also be equipped with the confidence, tools, and institutional support to translate this knowledge into clinical decision-making that prioritizes responsible antibiotic use. Likewise, microbiological research that identifies effective drug combinations or alternative agents offers promise for expanding the therapeutic arsenal in situations where resistance limits conventional options.

As AMR continues to evolve, further interdisciplinary research will be needed to test stewardship interventions, evaluate real-world outcomes, and ensure that dental healthcare systems contribute constructively to global efforts against antimicrobial resistance.

## References

[1] World Health Organization. Global Antibiotic Resistance Surveillance Report 2025: WHO Global Antimicrobial Resistance and Use Surveillance System (GLASS). Geneva: World Health Organization (2025). Available online at: https://www.who.int/publications/i/item/9789240116337 (Accessed February 10, 2026).

[2] Bessa LJ Botelho J Machado V Alves R Mendes JJ. Managing oral health in the context of antimicrobial resistance. Int J Environ Res Public Health. (2022) 19:16448. 10.3390/ijerph192416448 36554332 PMC9778414

[3] FDI World Dental Federation. Antibiotic stewardship in dentistry. (2020). Available online at: https://www.fdiworlddental.org/antibiotic-stewardship-dentistry (Accessed February 10, 2026).

[4] Teoh L Thompson W Suda K. Antimicrobial stewardship in dental practice. J Am Dent Assoc. (2020) 151(8):589–95. 10.1016/j.esmoop.2020.04.023 32718488

[5] D'Ercole S Parisi P D'Arcangelo S Lorusso F Cellini L Dotta TC Correlation between use of different type protective facemasks and the oral ecosystem. BMC Public Health. (2023) 23:992. 10.1186/s12889-023-16936-6 37828542 PMC10571399

